# The diagnostic value of the olfactory evaluation for congenital hypogonadotropic hypogonadism

**DOI:** 10.3389/fendo.2022.909623

**Published:** 2022-09-16

**Authors:** Bingqing Yu, Kepu Chen, Jiangfeng Mao, Bo Hou, Hui You, Xi Wang, Min Nie, Qibin Huang, Rui Zhang, Yiyi Zhu, Bang Sun, Feng Feng, Wen Zhou, Xueyan Wu

**Affiliations:** ^1^ National Health Commission, Key laboratory of Endocrinology, Department of Endocrinology, Peking Union Medical College Hospital, Peking Union Medical College, Chinese Academy of Medical Sciences, Beijing, China; ^2^ Department of Psychology, University of Chinese Academy of Sciences, Beijing, China; ^3^ State Key Laboratory of Brain and Cognitive Science, Chinese Academy of Sciences, Beijing, China; ^4^ Center for Excellence in Brain Science and Intelligence Technology, Institute of Psychology, Chinese Academy of Sciences, Beijing, China; ^5^ Department of Radiology, Peking Union Medical College Hospital, Peking Union Medical College, Chinese Academy of Medical Sciences, Beijing, China

**Keywords:** hypogonadotropic hypogonadism, Kallmann syndrome, Chinese olfactory function test, MRI, olfactory bulb

## Abstract

**Objective:**

The aim of this study was to evaluate the diagnostic accuracy of different olfactory evaluation tools in congenital hypogonadotropic hypogonadism (CHH) patients.

**Methods:**

Seventy-one CHH patients were prospectively recruited at Peking Union Medical College Hospital between November 2020 and July 2021. The Chinese Olfactory Function Test (COFT) and Self-reported Olfactory Scale (SROS) were adapted as the subjective tools for the evaluation of olfactory function, and magnetic resonance imaging of olfactory apparatus (MRI-OA) was the objective tool. The olfactory bulb volume (OBV) and the olfactory sulcus depth (OSD) were quantified.

**Results:**

Based on the COFT, 36 patients were categorized as having normosmic CHH (nCHH), and the other 35 patients were categorized as having Kallmann syndrome (KS). Among nCHH patients, 35 patients were classified as having normal olfaction and 1 patient had abnormal olfaction by SROS. For KS patients, there were 30 patients grouped into abnormal olfaction, while 5 patients had normal olfaction by SROS. For MRI-OA, 67% (18/27) of nCHH patients showed normal olfactory apparatus, and 33% (9/27) showed bilateral or unilateral olfactory bulb aplasia or hypoplasia. Among KS patients, 96% (27/28) of patients showed bilateral olfactory bulb hypoplasia or aplasia, and 4% (1/28) of patients showed normal olfactory apparatus. All six patients with unilateral olfactory bulb aplasia and three patients with bilateral olfactory bulb aplasia showed normal olfactory function. The accuracy of the SROS in the diagnosis of nCHH and KS was 91.5%, with a sensitivity of 0.857 and a specificity of 0.972, while the accuracy of MRI-OA is 92.7%, with a sensitivity of 0.964 and a specificity of 0.889.

**Conclusion:**

SROS and MRI-OA both showed high accuracy to distinguish between KS and nCHH. The abnormal structure of the olfactory apparatus was relatively common in nCHH patients. CHH patients with unilateral olfactory bulb aplasia dysplasia usually had normal olfaction. Normal olfaction without apparent olfactory bulbs is rare but occurred in male CHH patients.

## Introduction

Congenital hypogonadotropic hypogonadism (CHH) is a disorder with a prevalence of 1:4,000–10,000, which is caused by the defect in gonadotropin-releasing hormone (GnRH) release, action, or both (1). It is characterized by absent or incomplete sexual maturation. In addition, some patients with CHH exhibit other developmental defects, including anosmia, synkinesia, cleft lip/palate, sensorineural hearing loss, renal agenesis, syndactyly, or brachydactyly (2).

During the embryonic period, GnRH neurons were accompanied by olfactory axons migrating from the olfactory placode to the hypothalamus (3). The failure of the migration process results in hypogonadotropic hypogonadism and hypoplasia or aplasia of the olfactory system, namely, Kallmann syndrome (KS), which accounts for about 50%–60% of CHH (4). CHH patients with normal olfactory function are normosmic CHH (nCHH).

The evaluation tools of the olfactory function include objective tools and subjective tools (5). Olfactory event-related potentials (OERPs) are considered as the gold standard method to objectively evaluate olfactory function. However, this technique is complex, time-consuming, and not routinely performed in clinical practice (6). Psychophysical evaluation of the olfactory function is a low-cost and simple way, such as the University of Pennsylvania Smell Identification Test (UPSIT, USA) and the Sniffin’ Sticks (SS, Germany), which have been widely used in clinical and research settings (7). However, in mainland China, the olfactory function is scantly tested in clinical practice, partially due to the lack of a culturally appropriate smell identification test. Some odors in UPSIT and SS are unfamiliar to most Chinese (e.g., sauerkraut, raspberry, and rum), which leads to inaccurate results. In 2019, Wen Zhou et al. developed the Chinese smell identification test (CSIT) through two experiments. In the first experiment, the researchers obtained 45 odorants, which were the highest ranked odorants based on the familiarity and identifiability of 105 odor items in 296 participants. In the second experiment, 46 participants were shown the 45 odorants and were asked to make a choice of each odorant’s name from a list of four options. The odors misidentified by more than 30% of participants were excluded, and finally, the 40 most familiar odors of Chinese people were chosen in the CSIT (8). The researchers also verified that the identification accuracy of CSIT was higher than that of the UPSIT or SS-16 (8). It provides an effective tool for the assessment of olfactory function in the Chinese population. CSIT has been studied in Parkinson’s disease (PD) (9), but it has not been used in CHH in any studies so far.

In this study, we evaluated the olfactory function of 71 Chinese CHH patients using the Chinese Olfactory Function Test (COFT) and analyzed the influence factors of the olfactory function in CHH patients.

## Materials and methods

### Patients

Seventy-one CHH patients were prospectively recruited at Peking Union Medical College Hospital between November 2020 and July 2021. The diagnosis of CHH was based on the following criteria (10): (1) clinical signs or symptoms of hypogonadism; (2) low or normal gonadotropins, along with (a) serum testosterone levels below 100 ng/dl in men and (b) primary amenorrhea and estradiol levels below 20 pg/ml in women; (3) otherwise normal biochemical tests of anterior pituitary function; and (4) normal imaging (MRI) of the hypothalamic and pituitary area. Exclusion criteria were as follows: (1) tumor, surgery, and/or radiation in the sellar region; (2) traumatic brain injury; and (3) severe systemic disease. The study protocol was reviewed and approved by the ethics committee of the Peking Union Medical College Hospital in China. Written informed consent was obtained from all participants.

A detailed medical history, including the presence of cryptorchidism and other malformations, was taken. Serum total testosterone (ng/ml), serum FSH (U/L), and LH (U/L) were measured. Bilateral testis volumes (ml) were evaluated by the Prader orchidometer methodology.

### Olfaction

Olfactory function was estimated with the Self-reported Olfactory Scale (SROS) and a qualitative olfaction test using the COFT. SROS used a five-point scale and was grouped into five categories, namely, good, above average, average, below average, and poor. Patients in the “good”, “above average”, and “average” categories were classified as having normal olfactory function, while patients in the “below average” and “poor” categories were classified as having an abnormal olfactory function (9). The COFT includes three tests, the Chinese Smell Threshold Test (CSTT), the Chinese Smell Discrimination Test (CSDT), and the Chinese Smell Identification Test (CSIT) (8). According to the total score of the three tests Threshold-Discrimination-Identification (TDI), patients were divided into a normal olfactory function group (TDI ≥ 29.25) and an abnormal olfactory function group (TDI < 29.25). The factors that may affect olfactory function, including the diseases related to the olfactory system, and the history of smoking and drinking were collected in detail before COFT.

### MRI technique

Patients underwent the MRI of the olfactory apparatus (MRI-OA) on a 3-T MR system (Sonata Vision; Siemens, Germany) using the eight-channel coil. All the MRIs were reported by a single, experienced radiologist blindfolded for clinical findings. Volumes of the right and left olfactory bulbs (OBs) were determined using MRI scans of the olfactory apparatus and a standardized protocol for OB analysis. OB volumes (OBVs) were calculated by planimetric manual segmentation technique (surface in mm^2^), and all surfaces were added and multiplied by 3.6 because of the 3-mm slice thickness and the 0.6-mm gap to obtain a volume in cubic millimeters. If no bulb was identified on MRI, the volume was considered as zero. The olfactory sulcus depth (OSD) was measured using the coronal images. An immeasurable or absent sulcus was considered as zero.

### Statistical analysis

IBM SPSS Statistics 21.0 was used for data analysis. Normal distribution data were expressed as the mean ± SD and non-normal distribution data were listed as the median (quartiles). For normally distributed data, an independent-sample *t*-test was used for group comparisons. Comparisons of proportions were conducted using either the chi-square test or the Fisher exact test as indicated. Linear regression models and the multivariate linear regression model were built to analyze the determinants of olfactory function in CHH patients. Receiver operating characteristic (ROC) curves and area under the ROC curve (AUC) analysis were created to assess the ability of SROS and MRI-OA in evaluating olfactory function. Significance was accepted if *p* ≤ 0.05.

## Results

### Basic clinical characteristics

A total of seventy-one CHH patients were evaluated, including 69 male patients and 2 female patients. The average age of these patients was 23.6 ± 5.8 years. Fourteen (33%, 14/43) patients had cryptorchidism and two patients had unilateral renal agenesis (5%, 2/40). Hearing defect and syndactyly were shown in one patient (1%, 1/71). The ratio of the patients with smoking, drinking, and nose diseases were 25%, 28%, and 31%. All patients had low serum testosterone, estradiol, LH, and FSH levels, which indicated the diagnosis of CHH (**Table 1**).

**Table 1:** Baseline clinical characteristics of the included CHH patients.

	All patients	Untreated (*n* = 28)	Treated (*n* = 43)	*p*-value	nCHH (*n* = 36)	KS (*n* = 35)	*p*-value
Age, years	23.6 ± 5.8	22.2 ± 5.4	24.6 ± 5.8	0.085	23.2 ± 4.7	24.0 ± 6.7	0.600
BMI, kg/m^2^	25.1 ± 4.2	24.6 ± 4.5	25.5 ± 4.0	0.415	25.4 ± 4.4	24.9 ± 4.0	0.603
Follow-up time					46.4 ± 26.7	54.0 ± 38.9	0.587
TV, ml	6 ± 5	4 ± 3	8 ± 6	0.001	7.4 ± 6.1	4.7 ± 4.4	0.036
LH, U/L	1.15 ± 2.26	0.54 ± 0.55	1.6 ± 2.88	0.031	0.96 ± 1.50	1.35 ± 2.87	0.483
FSH, U/L	2.33 ± 3.65	1.09 ± 1.07	3.23 ± 4.54	0.007	2.18 ± 2.83	2.49 ± 4.40	0.804
T, ng/ml	1.55 ± 1.52	0.50 ± 0.41	2.25 ± 1.58	0	1.49 ± 1.58	1.61 ± 1.46	0.746
E2, pg/ml	20.31 ± 11.66	15.52 ± 1.40	23.81 ± 14.38	0.001	21.12 ± 14.46	19.4 ± 7.07	0.561
Cryptorchid, *n* (%)	14 (33%)	5 (31%)	9 (33%)	0.888	4 (19%)	10 (45%)	0.065
Unilateral renal agenesis, *n* (%)	2 (5%)	2 (11%)	0 (0%)	0.219	0 (0%)	2 (10%)	0.488
Hearing defect, *n* (%)	1 (1%)	1 (3%)	0 (0%)	0.394	0 (0%)	1 (3%)	0.493
Syndactyly, *n* (%)	1 (1%)	0 (0%)	1 (2%)	1	0 (0%)	1 (3%)	0.493
Smoking, *n* (%)	18 (25%)	7 (25%)	11 (26%)	0.956	8 (22%)	10 (29%)	0.539
Drinking, *n* (%)	20 (28%)	8 (29%)	12 (28%)	0.951	9 (25%)	11 (31%)	0.605
Nasal disease, *n* (%)	22 (31%)	5 (18%)	17 (40%)	0.054	12 (33%)	10 (29%)	0.664

TV, the average volume of right and left testis; LH, luteinizing hormone; FSH, follicle-stimulating hormone; T, testosterone; E2, estradiol.

Among these patients, 28 patients were untreated and 43 patients were treated. Twenty-five patients had gonadotropin therapy, nine patients had hormone replacement therapy, and seven patients had pulsatile gonadotropin-releasing hormone therapy. The level of serum LH, FSH, T, and E2 was higher in patients who were treated than in the untreated patients. There is no difference in age, BMI, the prevalence of cryptorchid, unilateral renal agenesis, hearing defect and syndactyly, and the ratio of the patients with smoking, drinking, and nose diseases between the two groups of patients (**Table 1**).

### Olfactory evaluation

SROS showed 3 patients in the good category, 13 patients in the above-average category, 24 patients in the average category, 3 patients in the below-average category, and 28 patients in the poor category. Overall, 40 patients were rated as normal olfactory function and 31 patients were rated as abnormal olfactory function.

COFT showed that 36 (51%) patients had normal olfactory function, which was categorized as normosmic congenital hypogonadotropic hypogonadism (nCHH). Thirty-five other patients (49%) had abnormal olfactory function, which was categorized as KS (**Table 2**).

**Table 2:** The olfactory function of CHH patients.

		All patients	Untreated (*n* = 28)	Treated (*n* = 43)	*p*-value	nCHH (*n* = 36)	KS (*n* = 35)	*p*-value
SROS	Good, *n* (%)	3 (4%)	2 (7%)	1 (2%)	0.558	3 (8%)	0 (0%)	0.248
	Above average, *n* (%)	13 (18%)	6 (21%)	7 (16%)	0.583	12 (33%)	1 (3%)	0.001
	Average, *n* (%)	24 (34%)	8 (29%)	16 (37%)	0.452	20 (56%)	4 (11%)	0.000
	Below average, *n* (%)	3 (4%)	1 (4%)	2 (5%)	0.999	1 (3%)	2 (6%)	0.980
	Poor, *n* (%)	28 (39%)	11 (39%)	17 (40%)	0.983	0 (0%)	28 (80%)	0.000
COFT	CSTT	9.58 ± 5.21	9.76 ± 5.31	9.47 ± 5.24	0.857	11.59 ± 3.88	3.90 ± 4.25	0.000
	CSDT	9.87 ± 3.66	9.93 ± 3.42	9.83 ± 3.85	0.924	11.41 ± 1.54	5.09 ± 4.23	0.000
	CSIT	11.85 ± 3.97	12.29 ± 3.26	11.59 ± 4.37	0.565	13.79 ± 1.37	6.33 ± 3.75	0.000
	TDI	21.44 ± 17.23	21.68 ± 17.53	21.29 ± 17.23	0.927	36.75 ± 4.68	5.70 ± 9.23	0.000
	NOF, *n* (%)	36 (51%)	15 (54%)	21 (49%)	0.697			
	AOF, *n* (%)	35 (49%)	13 (46%)	22 (51%)				
MRI-OA	OBV, mm^3^	19.66 ± 23.99	18.62 ± 22.30	20.34 ± 25.38	0.797	37.58 ± 20.64	2.40 ± 10.72	0.000
	OSD, mm	4.70 ± 3.43	4.85 ± 3.65	4.59 ± 3.32	0.787	6.82 ± 2.31	2.64 ± 3.07	0.000
	BOA, *n* (%)	30 (55%)	11 (52%)	19 (56%)	0.800	3 (11%)	27 (96%)	0.000
	UOA, *n* (%)	6 (11%)	3 (15%)	3 (9%)	0.852	6 (22%)	0 (0%)	0.027
	Normal MRI-OA, *n* (%)	19 (34%)	7 (33%)	12 (35%)	0.882	18 (67%)	1 (4%)	0.000

SROS, self-reported olfactory scale; COFT, Chinese Olfactory Function Test; CSTT, Chinese smell threshold test; CSDT ,Chinese smell discrimination test; CSI,: Chinese smell identification test; NOF, normal olfactory function; AOB, abnormal olfactory function; MRI-OA, MRI of olfactory apparatus; OBV, average olfactory bulb volume; OSD, average olfactory sulcus depth; BOA, bilateral olfactory bulb aplasia; UOA, unilateral olfactory bulb aplasia.

Fifty-five patients underwent on the MRI of the olfactory apparatus. It showed that six patients had bilateral OB hypoplasia with or without olfactory sulcus aplasia. Twenty-four patients had bilateral OB aplasia. Unilateral OB aplasia and hypoplasia were shown in three patients. Nineteen patients (29%) had normal olfactory apparatus (**Table 2**).

### Olfactory function analysis of normosmic congenital hypogonadotropic hypogonadism and Kallmann syndrome

According to the evaluation results of COFT, patients were divided into nCHH and KS patients. There was no difference in the baseline clinical data between nCHH and KS (**Table 1**). The incidence of cryptorchid, hearing defect, renal agenesis, and syndactyly was higher in KS than in nCHH patients, but there is no significant statistical difference (**Table 1**).

For SROS, 3 nCHH patients were in the good category, 12 patients were in the above-average category, 20 patients were in the average category, and 1 patient was in the below-average category. Among KS patients, there were 2 patients in the below-average category, 28 patients in the poor category, 4 patients in the average category, and 1 patient in the above-average category.

For MRI-OA, 67% (18/27) of nCHH patients showed normal olfactory apparatus, 22% (6/27) showed unilateral OB aplasia or hypoplasia, and 11% (3/27) showed bilateral OB hypoplasia or aplasia. Among KS patients, 96% (27/28) of patients showed bilateral OB hypoplasia or aplasia, and 4% (1/28) showed normal olfactory apparatus. The nCHH patients had larger OBVs and longer OSDs than KS patients (37.58 ± 20.64 *vs*. 2.40 ± 10.72, *p* = 0.000; 6.82 ± 2.31 *vs*. 2.64 ± 3.07, *p* = 0.000) (**Table 2**).

**Figure 1:**
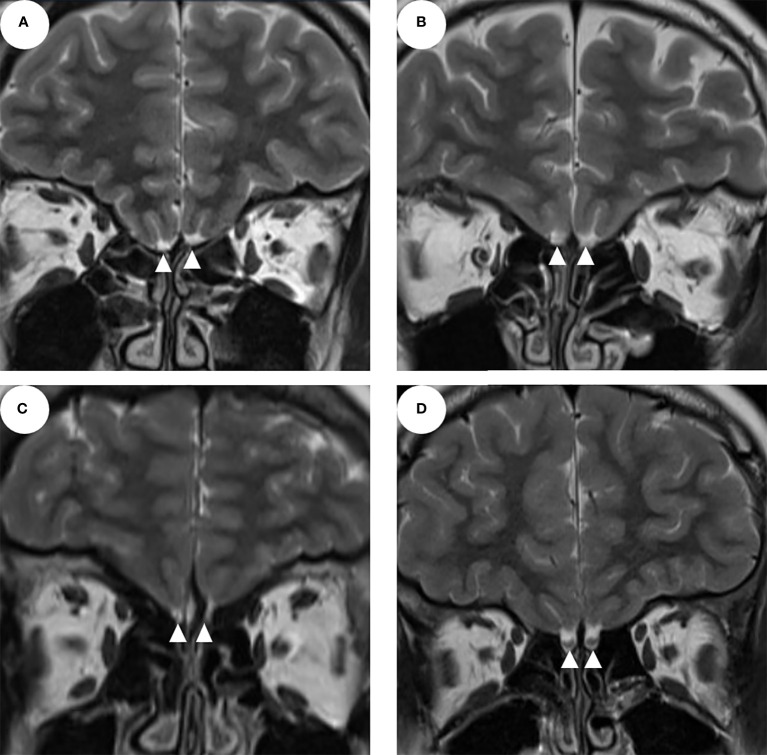
The MRI of the olfactory apparatus (**A–C** show the bilateral olfactory bulb and/or sulcus aplasia; **D** shows normal olfactory bulbs and sulcus; the white triangles indicate the olfactory bulbs).

Interestingly, six patients with unilateral olfactory apparatus aplasia and three patients with bilateral OB aplasia had normal olfactory function (**Figures 1A–C**), which were categorized as nCHH patients. One of the KS patients showed normal olfactory apparatus (**Figure 1D**).

### Determinant analysis for olfactory function in CHH patients

Univariate linear analysis used the TDI as the dependent variable and other factors (including age, BMI, follow-up time, T, E2, smoking, drinking, nasal disease, SROS, OBV, and OSD) as independent variables. It showed that the higher SROS, the larger OBV, and the longer OSD were associated with the higher TDI, while age, BMI, follow-up time, the level of serum T, smoking, drinking, and nasal disease had no significant correlation with olfactory function (**Table 3**).

**Table 3:** Univariable and multivariable linear regression analysis for determinants of olfactory function in CHH patients.

	Univariable Analysis			Multivariable Analysis *R* ^2^ = 0.752		
Variables	*t*	*R* ^2^	*p-*value	*t*	*β*	*p-*value
Age	−0.608	0.005	0.545	−0.943	−0.335	0.356
BMI	0.872	0.011	0.387			
Follow-up time	−1.021	0.025	0.314	1.186	0.093	0.249
T	−0.173	0.000	0.863	−1.660	−1.881	0.112
E2	0.771	0.009	0.444	1.468	0.175	0.157
Smoking	−0.275	0.001	0.784			
Drinking	−0.253	0.001	0.801			
Nasal disease	0.533	0.004	0.595			
SROS	16.609	0.800	0.000			
OBV	7.993	0.547	0.000	2.669	0.293	0.014
OSD	7.024	0.482	0.000	−0.943	−0.335	0.356

SROS, self-reported olfactory scale; OBV, average olfactory bulb volume; OSD, average olfactory sulcus depth.

The multivariate linear regression analysis included age, BMI, follow-up time, T, E2, OBV, and OSD. The results showed that OBV and OSD were independent factors for olfactory function (**Table 3**).

### Comparison of the accuracy of SROS and MRI-OA in diagnosing KS

**Figure 2:**
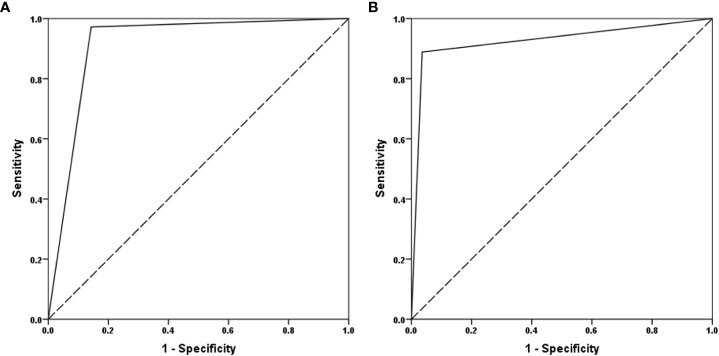
The ROC curve of SROS and MRI-OA. **(A)** MRI-OA; **(B)** SROS (SROS, self-report olfactory scale; MRI-OA, MRI of olfactory apparatus).

The COFT was used as an objective instrument to diagnose KS. The ROC curve analysis, using the SROS and MRI-OA rating as predictors for olfaction, revealed an area under the curve (AUC) of 0.915 and 0.927, respectively (**Figure 2**). The SROS predicted the nCHH and KS patients based on the COFT with a sensitivity of 0.857 and a specificity of 0.972. The MRI-OA predicted the nCHH and KS patients with a sensitivity of 0.964 and a specificity of 0.889.

## Discussion

In this study, we firstly used the COFT to evaluate the olfactory function of 71 CHH patients. Thirty-six patients were categorized as nCHH and the other 35 patients were categorized as KS. The CSTT, CSDT, and CSIT scores of nCHH patients were higher than those of KS patients.

Although olfactory event-related potentials (OERPs) are considered as the gold standard of olfactory function evaluation, they are rarely used in clinical practice because they are complex and time-consuming (11). The OB plays a relay station role in the processing of olfactory information. It receives the input signal from the olfactory epithelium and outputs it to the olfactory cortex (12). The OB is a neuronal structure of the forebrain and is easily detected on coronal weighted MR images (13). Studies have identified the positive correlation between electrophysiological and morphological changes of the olfactory pathway and psychophysical testing of olfactory functions in patients with olfaction loss, which means the lower amplitude in OERPs, the smaller OBV, the lower olfactory test score (14, 15). Thus, MRI of the olfactory apparatus is an objective method to evaluate the olfactory function.

In 1987, Klingmüller et al. (16) firstly used magnetic resonance imaging (MRI) to investigate alterations in structures related to olfactory function and found that in all four KS patients, the olfactory sulci are either absent or hypoplasic. Since then, other studies confirmed that MR can detect the absence of OBs and tracts in patients with KS, and it could be an objective method to evaluate the olfactory function of CHH patients (13, 17). Studies have found that the OBV is significantly associated with olfactory function scores (18–21), which is consistent with our study.

Studies have reported that the main characteristic of the olfactory apparatus of Kallmann patients was the absence of OBs and tracts, which were usually bilateral or unilateral (22–24). In our study, 27 out of 28 KS patients showed bilateral OB aplasia with or without olfactory sulcus aplasia. One KS patient showed normal olfactory apparatus. As reported in previous studies, normal olfactory apparatus can be found in patients with confirmed olfaction disorder (23).

However, unlike previous studies, our study found that the six CHH patients with unilateral olfactory apparatus dysplasia were on the left side and their olfactory function was normal and assigned to nCHH patients. It is speculated that it may be related to the compensation of olfactory function. Interestingly, three nCHH patients showed bilateral OB aplasia in our study, which has been considered as normal olfaction without apparent olfactory bulbs (NOWAOB). In 2019, Weiss et al. (25) first reported this phenomenon. They found 2 out of 1,113 patients combined with NOWAOB, and the 2 patients were female and left-handed. Thus, they reported that the NOWAOB was associated with left-handed women, while in our study, three right-handed male patients showed NOWAOB, which expanded the phenotype of NOWAOB. The detailed mechanism of NOWAOB was unclear. It is speculated that it may be related to OBs having migrated to a different brain location or OBs are just too tiny for the current MRI technology to detect since the slice thickness of MRI is 3 mm. Furthermore, a previous study had reported that CHH patients with normal olfactory function but abnormal olfactory apparatus showed declined olfactory function with age (26). The study showed the presence of a possible age effect on the olfactory function. Therefore, the olfactory function should be evaluated for our three nCHH patients with NOWAOB in their later age.

It is worth noting that our study found that the olfactory pathway structure and olfactory function are not always consistent. However, studies have indicated the discordance of OBV and OERP. Patients with the agenesis of OB may have identifiable OERPs (27–29). Thus, the olfactory function of these patients may be underestimated for the lack of OERP test. Furthermore, nasal polyposis, asthma, septal deviation, turbinate hypertrophy, and allergic rhinitis are related to olfactory dysfunction (30). The related medical history was obtained by inquiring the patients enrolled in our study who did not have the rhinoscopy examination. This may also lead to underestimate the olfactory function (31), which is the limitation of our study.

The self-reported smell is the most commonly used method to evaluate olfactory function in clinical practice, although it is noteworthy that SROS may overestimate the olfactory function of CHH patients (19, 21). Our study showed that the accuracy of SROS in the diagnosis KS was about 91.5%. Similar to other studies (32), the results also showed that self-reported normal olfaction is more unreliable, since the sensitivity was 85.7% and the specificity was 97.2%. Our study showed that the accuracy of MRI in the diagnosis of KS was about 92.7%, the sensitivity was 96.4%, and the specificity was 88.9%, which is consistent with other studies that had reported the sensitivity was 76%–100% (33). Thus, our study revealed that SROS is more reliable in the diagnosis of KS and MRI is more reliable in the diagnosis of nCHH.

CHH is a genetic disease and more than 40 pathogenic genes of the disease have been identified (34, 35). Theoretically, nCHH and KS have different genetic backgrounds. The KS occurs due to the migration failure of GnRH neurons during the embryonic period, which causes GnRH deficiency and a defective sense of smell, while nCHH occurs due to isolated GnRH deficiency without olfactory involvement (36, 37). However, most of the known CHH-related genes can cause both KS and nCHH (38–41), which indicate that these genes may contribute to different processes of the pathogenesis of CHH. Sykiotis et al. reported two patients diagnosed as having KS and nCHH, but they had the same gene variants (*FGFR1* and R250Q) (42). Thus, the differential diagnostic value of gene sequencing technology in KS and nCHH needs to be further explored.

For clinical characteristics, KS and nCHH show differences in developmental abnormalities. Previous studies reported that these phenotypes are more common in KS than in nCHH (43). The incidence of cryptorchid, hearing defect, renal agenesis, and syndactyly in nCHH and KS was 20%–40% vs. 12.5%–75%, 0%–11.1% vs. 7%–37.5%, 0%–6% vs 8%–15%, and 0%–6% vs. 0%–12.5%, respectively (43–46). In our study, although there is no significant statistical difference, the incidence of these abnormalities was higher in KS than in nCHH, which is consistent with the previous study.

Some studies suggested that the sex hormone may have an influence on olfactory function. Kırgezen et al. (47) evaluated olfactory function in 70 patients with prostate cancer and found that testosterone level was significantly correlated with olfactory function score, and the score of patients with low testosterone level was lower than that of the control group. Lee et al. analyzed the olfactory function of 3,863 women participants. It showed that the incidence of olfactory dysfunction in postmenopausal women was higher than that in premenopausal women. The longer the breastfeeding period of premenopausal women, the higher the risk of olfactory dysfunction, while the younger the postmenopausal women start to menopause, the higher the risk of olfactory dysfunction. Therefore, the study suggested that olfactory dysfunction was associated with endogenous estrogen deprivation (48). In clinical practice, our team also found that some KS patients with hormone replacement therapy subjectively reported an improved sense of smell. However, in this study, the results showed no difference in olfactory function between treated and untreated CHH patients, and there is no significant correlation between treatment time and sex hormone levels and olfactory function. Because this is a cross-sectional study, prospective studies are needed to confirm the results.

In conclusion, our study evaluated the olfactory function of 71 CHH patients. The olfactory function of the CHH patients with unilateral olfactory apparatus dysplasia is usually normal and few CHH patients without apparent OBs had normal olfaction. These remind us that the abnormal structure of the olfactory apparatus does not always support the diagnosis of KS, which could be beneficial to the precise diagnosis and classification of CHH patients.

## Limitations

This study is a cross-sectional study, and prospective studies are needed to analyze the effect of hormone replacement therapy on olfactory function in patients with CHH. It is unable to analyze the influence of genes on olfactory function because of the lack of genetic test in these patients.

## Data availability statement

The original contributions presented in the study are included in the article/supplementary material. Further inquiries can be directed to the corresponding author.

## Ethics statement

This study was reviewed and approved by Peking Union Medical College Hospital. The patients/participants provided their written informed consent to participate in this study.

## Author contributions

XYW, JM and MN conceived and designed the study. BY conducted the COFT and wrote the paper. KC and WZ provided the COFT tool. BH, HY and FF collected the MRI data. XW, QH, RZ, YZ and BS helped collect the clinical data. All authors contributed to the article and approved the submitted version.

## Funding

This work was supported by the Natural Science Foundation of Beijing (Grant No. 7212080 and 7202151).

## Acknowledgments

We thank the subjects and their family members for their participation in this research study.

## Conflict of interest

The authors declare that the research was conducted in the absence of any commercial or financial relationships that could be construed as a potential conflict of interest.

## Publisher’s note

All claims expressed in this article are solely those of the authors and do not necessarily represent those of their affiliated organizations, or those of the publisher, the editors and the reviewers. Any product that may be evaluated in this article, or claim that may be made by its manufacturer, is not guaranteed or endorsed by the publisher.
